# COTPA Section 4 Compliance in Udupi City Municipal Corporation: An Exploratory Survey From Coastal Karnataka

**DOI:** 10.1155/ijod/1369614

**Published:** 2026-03-02

**Authors:** Nishu Singla, Vinissa Eliane Tauro, Krishanu Dutta, Ayan Pradhan, Ritesh Singla, Jishnu Pradeep, Bidisha Sarmah, Deepak Kumar Singhal

**Affiliations:** ^1^ Department of Public Health Dentistry, Manipal College of Dental Sciences, Manipal Academy of Higher Education, Manipal, India, manipal.edu; ^2^ Manipal College of Dental Sciences, Manipal Academy of Higher Education, Manipal, India, manipal.edu; ^3^ Department of Orthodontics and Dentofacial Orthopaedics, Manipal College of Dental Sciences, Manipal Academy of Higher Education, Manipal, India, manipal.edu

**Keywords:** compliance, COTPA, Manipal, public places, second-hand smoke, Section 4, tobacco regulation in India, Udupi

## Abstract

**Background:**

The Cigarettes and Other Tobacco Products Act (COTPA), 2003, prohibits smoking in public places under Section 4 to protect individuals from second‐hand smoke (SHS). However, compliance with this provision remains inconsistent across India.

**Aim:**

To assess compliance with Section 4 of COTPA in public places in the Udupi City Municipal Corporation (UCMC) of Karnataka.

**Methods:**

A cross‐sectional observational study assessed compliance with smoke‐free regulations in 189 public places selected by convenience sampling using a structured observational checklist. Compliance was categorised as full, partial, or non‐compliant. Data were analysed using descriptive statistics, chi‐square tests with Bonferroni‐adjusted post hoc analyses and binary logistic regression to estimate odds ratios (ORs) with 95% confidence intervals (CIs).

**Results:**

Of 189 sites, 38% were compliant (17.5% fully and 20.5% partially), while 62% were non‐compliant, primarily due to absent signage (48%); active smoking was observed at 14%. Place category significantly predicted compliance (*p*  < 0.001), with higher adherence in recreational venues (90%) and healthcare facilities (86.7%). Educational institutions, government offices, hospitality venues and hotels showed substantial non‐compliance (45.5%–51.8%) and significantly lower odds of compliance compared with healthcare facilities (ORs 0.09–0.15). Marketplaces exhibited very poor compliance (77.8% non‐compliant; OR = 0.03), while transportation hubs and religious sites were uniformly non‐compliant due to the absence of mandated signage.

**Conclusion:**

The findings highlight the need for stronger enforcement, routine monitoring and targeted awareness initiatives to improve compliance with smoke‐free legislation and protect public health.

## 1. Introduction

Tobacco use is a significant public health issue in India, contributing to numerous preventable diseases and deaths annually [1]. Moreover, involuntary exposure to second‐hand tobacco smoke poses a substantial and preventable health risk to non‐smokers [2]. The World Health Organization (WHO) emphasises the need for comprehensive smoke‐free environments to safeguard the public from SHS in all indoor public spaces and workplaces. Under Article 8 of the WHO Framework Convention on Tobacco Control (FCTC), India is committed to implementing effective measures that safeguard the public from tobacco smoke [3]. Aligned with these obligations, the Cigarettes and Other Tobacco Products Act (COTPA), enacted in 2003, is a comprehensive legislation regulating the production, supply and distribution of tobacco products in India. One of its key provisions, Section 4, prohibits smoking in public places to protect non‐smokers from SHS exposure [4].

However, despite COTPA’s comprehensive nature, compliance with its provisions has been inconsistent across regions and settings in India [5]. In a retrospective record review of reports and primary data sheets of the surveys conducted in 38 jurisdictions in India, it was found that only 51% public places followed the smoke‐free law [6]. In Karnataka, a study of four municipal corporations in the state reported widespread tobacco sales and poor compliance with several provisions of COTPA, including Sections 4, 5, 6 and 20 [7]. In South Bengaluru, only a small fraction of public places showed complete compliance with Section 4, with violations such as visible ashtrays and cigarette butts being common, along with many public places lacking appropriate awareness of the Act’s provisions [8]. While specific data on compliance with Section 4 of COTPA in the Udupi City Municipal Corporation (UCMC) is limited, existing literature from Karnataka suggests that compliance with the various provisions of the act varies significantly depending on local enforcement and public awareness [9–11].

The Udupi and Manipal regions, with their diverse populations and significant public spaces, including educational institutions, commercial establishments and healthcare facilities, present a unique opportunity to assess the effectiveness of tobacco control measures in protecting public health. Conducting a localised survey in these areas would provide valuable insights into the current compliance status and identify opportunities for improvement. Hence, the survey was conducted to assess compliance with Section 4 of the COTPA, 2003, in public places within the UCMC. The findings of this study will have significant implications for policymakers and public health advocates committed to strengthening tobacco control efforts at the local level.

## 2. Methodology

A cross‐sectional observational study was conducted in the UCMC of Karnataka, India, to assess compliance with Section 4 of the COTPA, which prohibits smoking in public places. The study involved non‐intrusive, passive observation of public behaviour in publicly accessible spaces without direct interaction with individuals, the collection of personal data or intervention [12]. However, the ethical clearance for this study was obtained from the Institutional Review Board and the Kasturba Hospital Ethics Committee, Manipal (IEC1/553/2025), ensuring compliance with institutional standards. The design served as an exploratory assessment to provide preliminary insights into the on‐ground implementation of COTPA.

A conservative sample‐size calculation for estimating a single proportion at 95% confidence with a compliance rate of 27.4% (*p* = 0.274) from a previous study indicates a required sample size of 189 locations for a precision of ± 6.4% [7]. It is appropriate for an exploratory assessment aiming for approximately 10% precision and produces valid preliminary estimates to guide local tobacco‐control action and future larger studies [13]. This sample size is adequate for generating reliable overall and sector‐wise descriptive estimates of compliance and for detecting moderate to large sector‐wise differences using inferential analyses. However, smaller differences between certain sectors, particularly those with fewer observed locations, may not have been detected.

Random sampling was not adopted in this study primarily due to the practical constraints and exploratory nature of the assessment. True random selection of public places requires a complete and well‐defined sampling frame of all public locations within the UCMC limits. Such an exhaustive, up‐to‐date listing does not exist, and generating one would have required substantial time, manpower and financial resources that were beyond the scope of this preliminary study. Hence, a pragmatic approach was adopted in selecting 189 survey locations, taking into account available time, resources and accessibility. Care was taken to ensure diverse representation across various public venues such as public offices like post offices, DC office, zila parishad office, municipality office, banks, police station, restaurants, canteens, cafes, shopping malls, shops, marketplaces, public transport stations—like railway station, bus stops and auto stands—educational institutions, hospitals, cinemas, parks, sports complexes and libraries (Table 1). This approach enabled a practical yet comprehensive understanding of COTPA compliance within the selected regions.

**Table 1:** Overall compliance status of public places with Section 4 of COTPA.

Compliance status	Number	Percentage (%)
Complaint (fully or partially)	72	38
Non‐compliant (smoking observed and/or no signage)	117	62
Total	189	100

Direct observations were made using a structured checklist to assess compliance with Section 4 of the COTPA (Table 2). Each site was observed for approximately 10–15 min, depending on crowd dynamics and accessibility. The checklist included evaluating the presence of clearly visible ’No Smoking’ signage, instances of active smoking, the availability and enforcement of designated smoking zones (where applicable) and whether staff or security personnel intervened when smoking was observed.

**Table 2:** Structured checklist for assessing compliance with Section 4 of COTPA.

Section	Item	Response options
A. General site information	Site/location name	—
	Type of public place (Table 3)	

B. Signage compliance	Presence of ’No Smoking’ signage	Present/absent
	Visibility of signage	Clearly visible/visible but obstructed/poorly visible/not visible
	Signage format compliance (COTPA rules)	Standard format/non‐standard

C. Observation of active smoking	Is any person actively smoking?	Yes/no
	Number of persons smoking	1/2–3/ >3

D. Enforcement/staff intervention	Staff or security present?	Yes/no
	If smoking occurred, did staff intervene?	Yes/no/no violation observed.
	Type of intervention	Verbal warning/asked to move/removal from premises/other

E. Designated smoking area (DSA)	DSA present?	Yes/no

F. Final assessment	Overall compliance status	Compliant/non‐compliant

**Table 3:** Compliance status of public places with Section 4 of COTPA.

Compliance status	Number	Percentage (%)
Fully compliant (standard signage + no smoking)	33	17.5
Partially compliant (non‐standard signage + no smoking)	39	20.5
Non‐compliant (no signage)	90	48
Non‐compliant (active smoking)	27	14
Total	189	100

**Figure 1:**
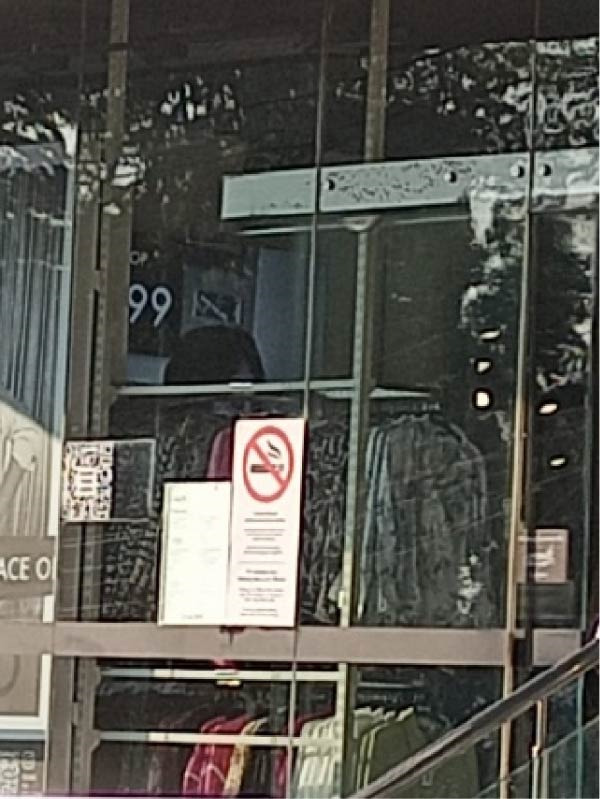
Fully compliant “No Smoking” signage prominently displayed at a conspicuous location.

**Figure 2:**
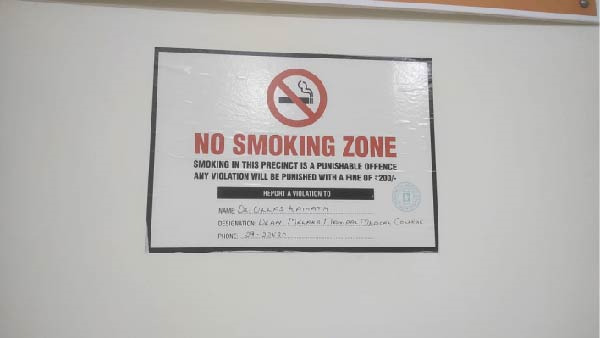
Fully‐compliant “No Smoking Zone” board displaying prescribed format, statutory warning, and authorized officer details.

**Figure 3:**
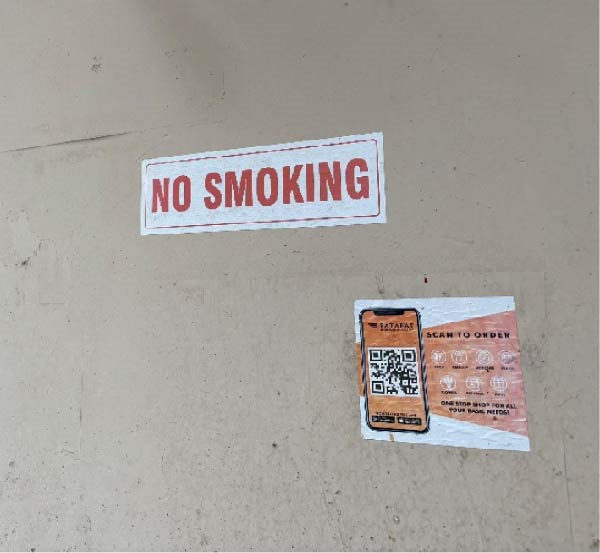
“No Smoking” sign displayed without mandatory COTPA‐specified format, statutory warning text, and authorized details.

**Figure 4:**
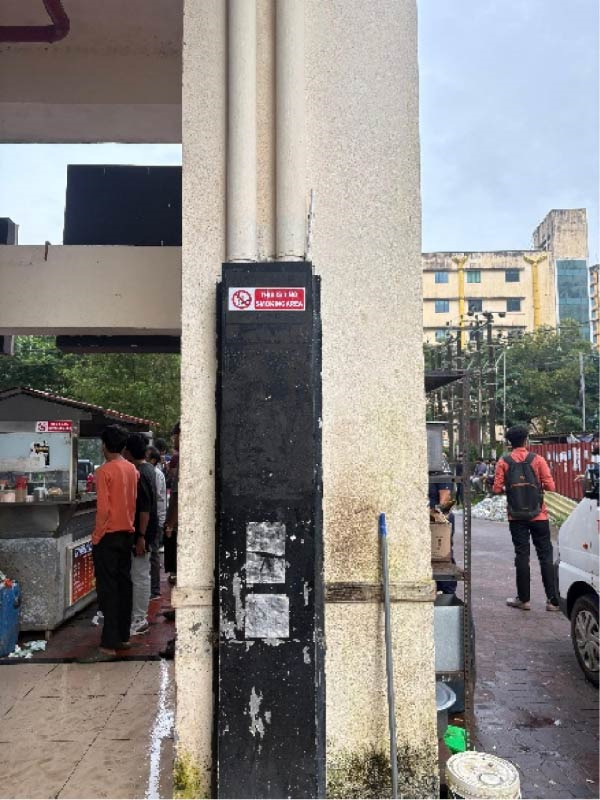
“No Smoking” signage displayed in a small, non‐standard format not complying with COTPA size and content specifications.

**Figure 5:**
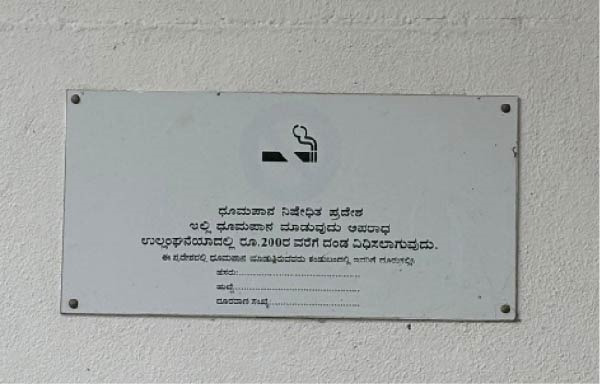
“No Smoking” signage lacking the prescribed red background, and authorized officer details as per COTPA specifications.

The locations were deemed non‐compliant if active smoking was observed in prohibited areas or if the required ’No Smoking’ signage was absent or not visible. Locations were classified as fully compliant if no active smoking was observed and if standard signage indicating smoking prohibition was present (Figures 1 and 2). Locations displaying non‐standard signage, but with no observed smoking, were considered partially compliant, acknowledging that signage deficiencies may reflect partial implementation or enforcement gaps rather than complete non‐adherence to smoke‐free regulations (Figures 3–5). However, for sector‐wise comparison, partially compliant locations were subsequently merged with the fully compliant category to facilitate meaningful comparisons across sectors and to avoid fragmentation of categories arising from small cell sizes.

As per the COTPA 2008 revisions, the standard ‘No Smoking’ signage must measure at least 60 cm × 30 cm with a white background, display the international ‘No Smoking’ symbol (a cigarette or beedi with smoke crossed by a red band) and carry the warning ’No Smoking Area – Smoking Here is an Offence’ in English or one Indian language. The signage must be prominently displayed at entrances and other visible locations within public places.

The data were entered into Microsoft Excel and subsequently analysed using IBM SPSS Statistics (version 26). Descriptive statistics were used to summarise the data, with frequencies and percentages presented to describe levels of compliance and non‐compliance across the observed locations. Associations between compliance status and type of public place were assessed using chi‐square tests. Post hoc analysis of significant chi‐square results was performed using adjusted standardised residuals to identify categories that contributed to the observed differences. To reduce the risk of Type I error arising from multiple comparisons, the Bonferroni correction was applied to the post hoc analyses, with the adjusted significance threshold set to the conventional alpha level (0.05) divided by the number of comparisons (36). A *p*‐value below the Bonferroni‐adjusted threshold was considered statistically significant (*p* = 0.0014).

As standardised residuals do not allow direct estimation of sector‐wise effect sizes, binary logistic regression was performed to compare compliance across place categories. Compliance status (yes/no) was entered as the dependent variable, and place category was included as a categorical independent variable. Odds ratios (ORs) with 95% confidence intervals (CIs) were calculated. Model fit was assessed using the Hosmer–Lemeshow goodness‐of‐fit test, which indicated an acceptable fit (*p*  > 0.05). Statistical significance was set at *p*  < 0.05.

## 3. Results

A total of 189 public places were assessed for compliance with Section 4 of the COTPA, which prohibits smoking in public places. Of these, 33 locations (17.5%) were classified as fully compliant, demonstrating both the absence of active smoking and the presence of standard ’No Smoking’ signage that met COTPA specifications (Figures 1and 2). An additional 39 locations (20.5%) were categorised as partially compliant, in which no active smoking was observed but the displayed signage was unclear or did not fully comply with COTPA requirements (Table 3 and Figures 3– 5). The overall compliance rate was 38% after partially compliant locations were merged with fully compliant sites, as the presence of a visible ‘No Smoking’ message was considered indicative of an attempt to comply with the law (Table 1). In contrast, 117 locations (62%) were non‐compliant (Table 1), with 90 sites (48%) lacking any ‘No Smoking’ signage and 27 sites (14%) recording instances of active smoking during the observation period (Table 3). In a few restaurants, designated smoking zones were identified, and in isolated instances, security personnel actively monitored the premises to prevent violations; however, these enforcement practices were sporadic and inconsistent. Compliance with Section 4 of COTPA differed significantly across categories of public places (*p*  < 0.001). Compliance was higher in healthcare facilities, with 33.3% fully compliant, 53.3% partially compliant and 13.3% non‐compliant. Recreational public venues, including cinemas, parks, sports complexes and libraries, showed the best overall compliance, with 70% fully compliant, 20% partially and 10% non‐compliant (Table 4).

**Table 4:** Compliance with Section 4 of COTPA across different types of public places.

Places	Total number	Compliance status	Number	Percentage (%)	*p*‐Value
Healthcare facilities	15	Fully complaint	5	33.3	**<0.001**
		Partially compliant	8	53.3	
		Non‐complaint	2	13.3	
Educational institutions	20	Fully complaint	4	20	
		Partially compliant	6	30	
		Non‐complaint	10	50	
Restaurants/canteens/cafes/bars	56	Fully complaint	7	12.5	
		Partially compliant	20	35.7	
		Non‐complaint	29	51.8	
Government/public offices like Udupi courts complex, post offices, banks, police station, DC office, zila parishad office and municipality office	11	Fully complaint	5	45.5	
		Partially compliant	1	9.1	
		Non‐complaint	5	45.5	
Market place/general stores/malls/shops	54	Fully complaint	7	13	
		Partially compliant	5	9.3	
		Non‐complaint	42	77.8	
Religious places	3	Fully complaint	0	—	
		Partially compliant	0	—	
		Non‐complaint	3	100	
Transport hubs: bus stops, auto stand, railway station	5	Fully complaint	0	—	
		Partially compliant	0	—	
		Non‐complaint	5	100	
Hotels/lodges	15	Fully complaint	5	33.3	
		Partially compliant	3	20.0	
		Non‐complaint	7	46.7	
Recreational public venues (cinemas, parks, library, sports complex)	10	Fully complaint	7	70	
		Partially compliant	2	20	
		Non‐complaint	1	10	
Total	189	—	189	100	—

*Note*: The *p*‐value was derived using a chi‐square test. Values in bold indicate highly statistically significant results (*p* < 0.001).

However, Educational institutions showed suboptimal adherence: 20% were fully compliant, 30% partially compliant and 50% non‐compliant. Government and public offices showed fully compliant rates of 45.5%, partially compliant rates of 9.1% and non‐compliant rates of 45.5%. Hospitality venues also performed inconsistently: restaurants, canteens, cafés and bars recorded 12.5% full compliance, 35.7% partial compliance and 51.8% non‐compliance, whereas hotels and lodges recorded 33.3% full compliance, 20% partial compliance and 46.7% non‐compliance (Table 4).

In contrast, poorly regulated, high‐footfall environments showed the weakest compliance. Marketplaces, general stores, malls and shops had very low adherence, with only 13% fully compliant, 9.3% partially compliant and 77.8% non‐compliant. Transportation hubs, including bus stops, auto stands and railway stations, were uniformly non‐compliant (100%). Although no active smoking was observed at religious sites, these locations were classified as 100% non‐compliant solely due to the absence of displayed signage (Table 4).

Post hoc analysis using adjusted standardised residuals revealed that only two place categories significantly contributed to the overall chi‐square association. Healthcare facilities demonstrated significantly higher compliance than expected (adjusted residual = 3.38, *p*  < 0.0014), whereas marketplaces and shopping complexes demonstrated significantly lower compliance than expected (adjusted residual = −3.98, *p*  < 0.0014). The remaining categories did not show statistically significant deviations after adjustment for multiple comparisons (Table 5).

**Table 5:** Post hoc Bonferroni‐adjusted standardised residuals identifying place categories significantly contributing to compliance differences.

Places	Total number	Compliance status	Number	Percentage (%)	AR	*p*‐Values
^a^Healthcare facilities	15	Complaint	13	86.7	3.38	**0.0007**
		Non‐complaint	2	13.3	−3.38	**0.0007**

Educational institutions	20	Complaint	10	50	0.48	0.6312
		Non‐complaint	10	50	−0.48	0.6312

Restaurants/canteens/cafes/bars	56	Complaint	27	48.2	0.58	0.5619
		Non‐complaint	29	51.8	−0.58	0.5619

Government/public offices like Udupi courts complex, post offices, banks, police station, DC office, zila parishad office and municipality office	11	Complaint	6	54.5	0.66	0.5093
		Non‐complaint	5	45.5	−0.66	0.5093

^a^Market place/general stores/malls/shops	54	Complaint	12	22.2	−3.98	**0.0001**
		Non‐complaint	42	77.8	3.98	**0.0001**

Religious places	3	Complaint	0	—	−1.58	0.1141
		Non‐complaint	3	100	1.58	0.1141

Transport hubs: bus stops, auto stand, railway station	5	Complaint	0	—	−2.05	0.0404
		Non‐complaint	5	100	2.05	0.0404

Hotels/lodges	15	Complaint	8	53.3	0.68	0.4965
		Non‐complaint	7	46.7	−0.68	0.4965

Recreational public venues (cinemas, parks, library, sports complex)	10	Complaint	9	90	2.94	0.0033
		Non‐Complaint	1	10	−2.94	0.0033

Total	189	—	189	100	—	—

*Note:* Values in bold indicate highly statistically significant results (*p* < 0.0014).

^a^Post hoc analysis using Bonferroni‐adjusted standardised residuals (*p* = 0.0014).

Binary logistic regression revealed that place category was a significant predictor of compliance status (Omnibus *χ* ^2^ = 44.183, *p*  < 0.001). Using healthcare facilities as the reference category, several types of public places demonstrated significantly lower odds of compliance with no‐smoking signage. Educational institutions had significantly reduced odds of compliance (OR = 0.154; 95% CI: 0.027–0.866; *p* = 0.034), as did restaurants, canteens, cafés and bars (OR = 0.092; 95% CI: 0.019–0.450; *p* = 0.003). Marketplaces, general stores, malls and shops exhibited the poorest compliance, with markedly lower odds compared to healthcare facilities (OR = 0.027; 95% CI: 0.005–0.142; *p*  < 0.001). Hotels and lodges also showed significantly reduced compliance (OR = 0.135; 95% CI: 0.022–0.816; *p* = 0.029). In contrast, differences observed for government and public offices (OR = 0.185; 95% CI: 0.028–1.239; *p* = 0.082) and recreational public venues (OR = 0.359; 95% CI: 0.048–2.683; *p* = 0.318) were not statistically significant. Complete non‐compliance in religious places and transportation hubs precluded reliable estimation of ORs (Table 6).

**Table 6:** Binary logistic regression analysis of factors associated with compliance with Section 4 of COTPA across public places.

Type of place	Odds ratio	(95% C.I)	*p*‐Value
Healthcare facilities	Ref	—	**0.002**
Educational institutions	0.154	(0.027–0.866)	**0.034**
Restaurants/canteens/cafes/bars	0.092	(0.019–0.450)	**0.003**
Government/public offices like Udupi courts complex, post offices, banks, police station, DC office, zila parishad office and municipality office	0.185	(0.028–1.239)	0.082
Market place/general stores/malls/shops	0.027	(0.005–0.142)	**0.000**
Religious places	0.000	—	0.999
Transport hubs: bus stops, auto stand, railway station	0.000	—	0.999
Hotels/lodges	0.135	(0.022–0.816)	**0.029**
Recreational public venues (cinemas, parks, library, sports complex)	0.359	(0.048–2.683)	0.318

*Note:* Values in bold indicate highly statistically significant results (*p* < 0.001).

## 4. Discussion

The assessment of 189 public places for compliance with Section 4 of the COTPA, which prohibits smoking in public areas, revealed suboptimal adherence in the Udupi Manipal region. Although compliance was noted in 38% of locations, only 17.5% were fully compliant, while the remaining 20.5% showed partial compliance due to failure to meet COTPA signage specifications. Non‐compliance was predominantly attributable to the absence of mandated signage (48%), while direct violations involving active smoking constituted a smaller proportion (14%). The instances of active smoking observed were concentrated in specific types of locations, primarily outside shopping complexes, a small eatery, a bus stop and a hotel parking area, where a driver was observed smoking. Repeated smoking was noted at certain sites outside shopping complexes, where groups of students, both males and females, reportedly congregated regularly and smoked in relatively large numbers. Several students seen smoking in Manipal, particularly outside the campus areas, reflect local sociocultural dynamics and indicate potential gaps in enforcement and awareness among persons responsible for monitoring these spaces, as well as the influence of social norms where smoking is perceived as trendy or socially acceptable among young adults in student‐dominated, transient environments. In contrast, the absence of active smoking in many other public places may be partly attributable to the nature of the locations included in the sample, such as hospitals, educational institutions, government offices, parks, temples and libraries, where smoking is socially unacceptable, institutionally restricted or structurally monitored.

Compared with other regions of India, the absence of active smoking in most locations and the proportion of signage displayed (38%) in this study are broadly comparable. A nationwide systematic review reported an overall compliance rate of 71.97% with Section 4, though only 42.3% of places displayed signage, highlighting that inadequate signage remains a widespread implementation gap nationally, rather than a setting‐specific failure [5]. Higher compliance has been reported from Punjab (83.8% across 6875 public places) and SAS Nagar, Mohali (92.3%), where 90% of public places displayed signage and 94.2% were free from active smoking [14, 15]. Similarly, in Alwar district, Rajasthan, 90% of public places displayed ‘No Smoking’ signage, with 99% meeting the COTPA specifications [16]. In contrast, moderate compliance has been documented in Chandigarh (signage in 54.6% of public places with 36.1% full compliance) and Shimla (48% compliance), while Puducherry showed relatively better overall compliance (67%) despite inconsistent signage display [17–19]. Markedly poor compliance was reported from South Bengaluru (30.9% of places with signage), Bengaluru (10.3% with signage) and Raipur (11.3% with signage) [8, 20, 21]. Another study from Bengaluru highlighted 67% of public places violated Section 4 [22]. The variability observed across regions highlights the role of local governance, enforcement prioritisation and stakeholder engagement in shaping compliance outcomes. The findings from Udupi–Manipal align more closely with settings characterised by moderate enforcement, reinforcing the need for targeted, context‐specific strengthening of signage enforcement rather than uniform national strategies.

High compliance with smoke‐free policies was observed in institutional settings, particularly healthcare facilities (86.7%) and recreational venues (90%). Healthcare settings often demonstrate better adherence to smoking prohibitions owing to their core mandate of health promotion [5, 6, 14, 15]. This pattern is further supported by inferential analyses in our study: healthcare facilities demonstrated significantly higher compliance than expected (adjusted residual = 3.38, *p*  < 0.0014), and logistic regression showed that institutional settings had higher odds of compliance compared to other public places. Routine monitoring mechanisms, clear role definition for security and administrative staff and reputational accountability together with social norms that discourage smoking in these public spaces, appear to reinforce adherence. Nevertheless, compliance across healthcare settings is not uniform. A study from Delhi Government hospitals reported substantially lower compliance: active tobacco use was observed in 40.6% of hospital buildings, 75.4% of public places outside the buildings and 21.4% of hospitals lacked COTPA‐compliant signage [23]. Similarly, a tertiary healthcare institution in Puducherry reported compliance as low as 23%, with active smoking documented in 52.5% of venues and signage present in just 20% [24]. Such lapses are concerning, as hospitals are expected to function as health‐promoting environments, and inadequate enforcement of smoke‐free policies can erode public trust and weaken the perceived commitment of these institutions to public health.

In contrast, educational institutions, government offices, restaurants and hotels demonstrated moderate compliance, with approximately half complying with the regulations. Although active smoking was infrequently observed on educational campuses, likely due to lower social acceptability of smoking in these environments and internal disciplinary mechanisms, only 50% of institutions displayed the mandated signage, indicating gaps in policy implementation. This figure is marginally lower than the 60% reported from a North Indian city but remains considerably higher than compliance reported in Chennai, where signage was observed in just 2.8% of schools and 14.8% of colleges [17, 25]. Even lower levels of compliance have been documented in urban Puducherry, where only 8% of public places and 1.7% of educational institutions met COTPA specifications [26]. Government offices in earlier studies have shown relatively better adherence than observed in the current findings, suggesting variability in administrative oversight and accountability across regions [17, 18]. Hospitality venues, including restaurants and hotels, consistently exhibit poorer compliance, likely due to commercial pressures, high customer turnover and limited perceived enforcement [6]. Studies from Chennai reported that 92.8% of restaurants lacked signage, while only 28% of hospitality establishments in Bengaluru and Dharwad displayed the required boards [25, 27].

The lowest levels of compliance were observed in high‐footfall, poorly regulated public environments such as marketplaces, shops and transport hubs. Marketplaces and shops showed poor compliance (22.2%), while transport hubs recorded complete non‐compliance. These places demonstrate significantly lower compliance than expected (adjusted residual = −3.98, *p*  < 0.0014), and logistic regression confirmed markedly reduced odds of compliance compared to healthcare facilities (OR = 0.027; 95% CI: 0.005–0.142; *p*  < 0.001). These settings pose unique challenges due to diffuse administrative responsibility, constant crowd movement and minimal visible authority or surveillance, often resulting in enforcement fatigue and role ambiguity among shop owners, transport staff and security personnel, who may not perceive smoke‐free enforcement as part of their formal responsibility. In the absence of clearly designated site‐level custodians and regular municipal inspections, compliance in such non‐institutional settings remains weak. The absence of signage and unchecked tobacco use in such spaces underscores critical gaps in enforcement and highlights the need for targeted enforcement strategies, clearer assignment of responsibility to local authorities and greater visibility of regulatory oversight to ensure effective implementation of smoke‐free legislation.

Overall, the observed compliance patterns reflect the interaction between local governance capacity, sociocultural norms and the unique urban educational landscape of the Udupi–Manipal region. Higher compliance in healthcare and recreational venues, and lower compliance in marketplaces and transportation hubs, likely reflect differences in governance structures: institutional settings operate under clearly defined administrative hierarchies, internal accountability mechanisms and routine oversight, whereas open public spaces are governed by diffuse or overlapping authorities. Several students were observed smoking in Manipal, a town characterised by a dense concentration of educational institutions, a large student population drawn from diverse geographic regions, and a predominantly transient residential profile. These sociocultural features, combined with local awareness gaps, likely contribute to altered risk perceptions, peer‐normalised smoking behaviours and reduced community‐level deterrence, particularly in semi‐public and commercial spaces surrounding campuses. Within the UCMC jurisdiction, implementation of Section 4 of the COTPA is primarily the responsibility of municipal authorities, the district health department, and designated enforcement agencies, including the police and authorised officers under the Act. The suboptimal overall compliance observed in this study suggests gaps in sustained monitoring, prioritisation of enforcement and clarity of accountability at the municipal level, particularly in non‐institutional public environments. For local policymakers and municipal authorities, these findings underscore the need to strengthen city‐level enforcement mechanisms, enhance interdepartmental coordination and tailor smoke‐free strategies to high‐risk settings such as student‐dominated commercial zones, marketplaces and transport hubs.

Beyond structural and administrative constraints, Limited awareness of smoke‐free provisions among persons in charge of public places, as reported in previous studies, likely contributes to inadequate signage display and passive tolerance of violations [8, 19, 28]. Evidence from Ramanagaram, Karnataka, indicates that only 36.5% of owners or persons in charge of public places were aware of smoke‐free legislation [29]. While a study from Jodhpur reported awareness of COTPA in only 45% of respondents, with just 30% knowing the appropriate authority to whom violations could be reported [30]. Such knowledge gaps, coupled with social acceptance of tobacco use, weak and inconsistent enforcement, variable political commitment, insufficient stakeholder engagement and inadequate training of law‐enforcement personnel, school administrators and vendors, further exacerbate non‐compliance [28, 31–33]. Studies from Karnataka also suggest that improved inter‐agency coordination can enhance adherence to smoke‐free regulations; however, in the absence of systematic monitoring and clearly defined accountability mechanisms, effective implementation remains limited [32, 33].

Within this context, the findings should be interpreted as a local situational analysis generating baseline evidence on compliance with Section 4 of the COTPA within the UCMC region. The exploratory approach was intended to document prevailing on‐ground conditions, identify public settings with prominent enforcement gaps, and highlight priority areas for regulatory attention. Rather than providing definitive prevalence estimates or causal explanations, the results offer context‐specific insights that can inform local enforcement strategies, stakeholder sensitisation and the planning of future analytical or interventional studies employing more rigorous sampling and longitudinal designs.

## 5. Limitations

Considering these context‐specific findings, certain methodological considerations should be acknowledged. The use of convenience sampling may have introduced selection bias and limited the generalizability of the findings beyond the Udupi Municipal Corporation setting; however, this approach was considered appropriate for an exploratory situational analysis aimed at generating baseline evidence and practical insights into on‐ground implementation of smoke‐free regulations. Although statistically significant associations between public place type and compliance status indicate that the study had adequate power to identify meaningful sector‐wise patterns, smaller differences between settings, particularly in settings with fewer observed locations, may not have been detected and should, therefore, be interpreted cautiously. Consequently, the findings should be viewed as preliminary and primarily intended to inform local tobacco control strategies and guide the design of future studies employing larger, more sector‐balanced samples. Additionally, as observations were passive and time‐bound, instances of active smoking or enforcement intervention may have been underestimated if violations occurred outside the observation periods. Some locations may, thus, have appeared compliant simply because no violation occurred during the observation window, despite the possibility of non‐compliance at other times.

## 6. Recommendations

Based on the observed compliance patterns, the findings support the need for context‐specific, tiered interventions within the Udupi–Manipal region. Given the complete non‐compliance documented in transport hubs and the poor compliance in marketplaces and shops, immediate measures should prioritise standardised placement of COTPA‐compliant ‘No Smoking’ signage in these high‐footfall settings, with clear assignment of responsibility to site custodians. As a medium‐term strategy, routine compliance audits led by municipal authorities and the local health department, integrated into existing inspection frameworks, could improve monitoring and accountability. In parallel, capacity‐building initiatives, including targeted orientation and sensitisation of shop owners, transport staff, security personnel and facility managers, are essential to improve awareness of legal obligations and reporting mechanisms. Together, these measures offer a pragmatic pathway for translating the study findings into actionable local policy and enforcement strategies.

## 7. Conclusion

Compliance with Section 4 of COTPA within the UCMC remains suboptimal, particularly in open, transient and commercial spaces, including marketplaces, transport hubs and areas around educational institutions. This variability is influenced by awareness gaps, socially accepted smoking behaviours and enforcement challenges, including fatigue and unclear responsibilities among staff and vendors. Institutional settings with clear authority structures and routine supervision demonstrate greater adherence, underscoring the importance of defined roles and sustained oversight. Strengthening enforcement, improving signage visibility, enhancing inter‐agency coordination and implementing targeted awareness initiatives, especially for young adults, are critical to advancing truly smoke‐free public environments.

## Funding

No funding was received for this manuscript.

## Conflicts of Interest

The authors declare no conflicts of interest.

## Data Availability

The data that support the findings of this study are available from the corresponding author upon reasonable request.
